# Methodological Perspectives on Cryptic Species Monitoring: Insights from Strictly Protected Lesser Blind Mole Rat

**DOI:** 10.3390/life16091408

**Published:** 2026-08-25

**Authors:** Marko Đokić, Vida Jojić, Pavle Lukić, Nataša Barišić Klisarić, Aleksandra Penezić, Vanja Bugarski-Stanojević

**Affiliations:** 1 Department of Genetic Research, Institute for Biological Research “Siniša Stanković”—National Institute of Republic of Serbia, University of Belgrade, 11108 Belgrade, Serbia; vjojic@ibiss.bg.ac.rs (V.J.); pavle.lukic@ibiss.bg.ac.rs (P.L.);; 2 Department of Evolutionary Biology, Institute for Biological Research “Siniša Stanković”—National Institute of Republic of Serbia, University of Belgrade, 11108 Belgrade, Serbia; natasa@ibiss.bg.ac.rs; 3 Faculty of Biology, University of Belgrade, 11158 Belgrade, Serbia; aleksandra.penezic@bio.bg.ac.rs

**Keywords:** chromosomal speciation, conservation, *Nannospalax*, non-lethal, karyotyping, ISSR-PCR, *Sry*

## Abstract

Cryptic biodiversity presents an ongoing challenge to the accuracy and effectiveness of biodiversity assessment and conservation. To address this, we developed a non-lethal genetic monitoring framework for the subterranean rodent, European lesser blind mole rat (BMR), *Nannospalax leucodon* species complex, which encompasses multiple chromosomally diversified and reproductively isolated cryptic-species and subspecies. Our workflow combines species and sex detection through: karyotyping, Inter-Simple Sequence Repeat (ISSR) PCR profiling, and *Sry*-based sex determination of 33 individual samples from five BMR cryptic-species collected at 25 localities in Serbia. As conventional karyotyping protocols for BMR required animal sacrifice, we developed the first non-lethal fibroblast culture-based karyotyping approach from BMR skin biopsy, producing high-quality metaphase chromosomes across five cryptic-species. Among twelve tested ISSR primers, three yielded reproducible, species-specific DNA profiles that resolved four of the five cryptic-species. The *Sry* assay accurately determined the sex of all examined individuals. Our findings demonstrate that integrating fibroblast culture-based karyotyping, with fast, cost-effective ISSR-PCR species identification, and *Sry*-based sex determination, provides a reliable approach for identifying and monitoring cryptic BMR species without sacrificing individuals. This framework has potential applications in conservation programmes and contributes to an integrative taxonomy approach essential for the study and protection of other cryptic taxa.

## 1. Introduction

Biodiversity is commonly defined as “the variability among living organisms including terrestrial, marine and other aquatic ecosystems and the ecological complexes of which they are a part”; this includes diversity within species, between species and of ecosystems [1,2]. Accurate biodiversity measurement, i.e., quantification, is fundamental for effective conservation, because species represent the basic units of biological diversity [3]. Species richness represents only one component of biodiversity, reflecting the number of species present in a given area. In contrast, biodiversity encompasses variation at multiple biological levels, including genetic diversity within species, diversity among species, and ecosystem diversity, highlighting the importance of conserving genetically distinct populations and subspecies [4]. Consequently, errors in species delimitation can have significant implications for both, biodiversity assessment and conservation planning.

A substantial part of global biodiversity constitute cryptic species, defined as two or more species that, due to their morphological similarity, have historically been or continue to be classified as a single species [3,5,6,7,8]. Because they are not easily differentiated, many cryptic species have remained, and in many cases still remain, undetected [9,10,11,12]. Their recognition is crucial for accurate biodiversity assessment and conservation [3]. A large proportion of them is endemic and vulnerable due to habitat loss and other anthropogenic environmental pressures, underscoring the need for their accurate identification, formal recognition and conservation.

Formal species designation is essential for the precise definition of conservation units, assessment of conservation status, and implementation of appropriate management actions [13]. Species designation is a multistep process that involves species identification, delimitation, and formal description, each presenting distinct conceptual and methodological challenges [14,15,16].

Conservation strategies are based on data collected in the field through various wildlife monitoring methods [17]. Genetic monitoring, as a powerful tool for the identification of cryptic species, represents an irreplaceable part of conservation programmes [18,19,20,21,22]. When surveying threatened taxa, it is important to use the least invasive sampling possible. In the past, sacrificing a certain number of animals for genetic research was routine; however, as this practice was ethically problematic and, in most cases, scientifically unnecessary, it has largely been superseded by advances in experimental design. In recent years, wildlife genetics has been revolutionized by the development of non-invasive and non-lethal sampling techniques which apply minimally invasive protocols, reduce stress and risk to individuals, while enabling reliable surveillance. Despite this progress, accessing high-quality tissue samples from free-ranging wild animals remains a considerable challenge, particularly when working with rare, endangered, or protected populations [18]. While completely non-invasive methods, such as environmental DNA (eDNA) analysis [19,20,22,23], exclude human-animal contact, they often fail to provide DNA/RNA quality necessary for certain comprehensive molecular-genetic studies. Furthermore, karyotype analyses are not possible without live cells, which require compulsory tissue sampling, followed by cell culturing.

The diversification of cryptic species can be driven by various mechanisms, including intensive chromosomal rearrangements [24] which are particularly common in rodents, especially within the families Muridae and Cricetidae [25]. In some cases, relatively recent chromosomal rearrangements have not yet resulted in genetic diversification or changes in morphological or physiological traits, but have instead led to the formation of distinct chromosomal forms [26]. A wide range of chromosomal forms can vary considerably even within the same species, which can complicate their identification. A notable example of this phenomenon is found in the genus *Nannospalax* (Palmer 1903)–the lesser blind mole rat (subfamily Spalacinae), which are known for their exceptional chromosomal diversity combined with morphological similarity [27,28].

The European lesser blind mole rat (BMR) *Nannospalax leucodon* (Nordmann 1840) superspecies comprises 25 different chromosomal forms (CFs) described so far with diploid chromosomal number (2n) from 46 to 58 and with different chromosomal morphology quantified through the number of chromosomal arms (NF), summarized in [27,28]. Seven were found to be reproductively isolated [28] and genetically well differentiated cryptic-species [29,30,31]. Although these taxa have traditionally been treated as subspecies under trinomial nomenclature, previous cytogenetic and molecular studies indicate that they represent distinct evolutionary lineages, corresponding to cryptic species within the *N. leucodon* complex [32]. Five of them are distributed in Serbia: *N. l. hungaricus* (Nehring 1898); *N. l. serbicus* (Méhely 1909); *N. l. montanoserbicus* (Savić and Soldatović 1974); *N. l. syrmiensis* (Méhely 1909) and *N. l. montanosyrmiensis* (Savić and Soldatović 1974). Despite a strictly protected status in Serbia [33], they have experienced a continuous decline in population number over the past six decades, primarily due to the loss of its natural habitats [30,31]. Recent studies highlight the urgent need for detailed monitoring and urgent conservation actions for protection of the two cryptic species that are critically endangered: *N. l. syrmiensis* and *N. l. montanosyrmiensis* [30,31,34].

For taxa with chromosomal variability and many chromosomal forms, including cryptic-species within the genus *Nannospalax*, accurate species identification frequently requires karyotyping [35]. Traditionally, preparation of metaphase chromosomes is performed on lymphocytes from bone marrow, a procedure that involves unnecessarily sacrificing [36]. Given their strictly protected status, the establishing primary fibroblast cell cultures using non-lethal sampling represents necessary and elegant alternative for obtaining karyotypes without the need for animal sacrifice.

Use of in vitro cell cultures in BMR’s karyotype assessment has been generally avoided for a long time because of reported infeasibility of BMRs fibroblasts attributed to the specialized features of these underground animals, such as exceptional concerted cell death, associated with their cancer resistance mechanisms [37]. Another possibility, the short-term culture of peripheral blood lymphocytes, commonly used in larger mammals, is generally not applicable to small mammals such as BMRs, because their limited blood volume precludes the collection of sufficient peripheral blood for lymphocyte culture. In addition to the absence of eyes, they lack a tail and auricles, and the blood vessels in their limbs are constricted to minimise injuries during burrowing.

Besides karyotyping, BMR cryptic species have been diagnosed using mitochondrial DNA barcoding [30,31,38], but there is a need for faster and lower-cost methods to improve the efficiency of BMR monitoring. Another genetic approach, Inter Simple Sequence Repeat PCR (ISSR-PCR), which amplifies genomic regions flanked by microsatellite sequences [39], has been demonstrated as a reproducible and reliable tool for identifying different plant [40] and animal species, such as fishes, rodents, and bats [41,42,43]. The product of this PCR-based method is a collection of dominant DNA markers within suitable size ranges, i.e., an ISSR DNA fingerprint. The main advantage is rapid, low cost, multi-locus screening of the whole genome and the production of species-specific profiles without prior sequence knowledge [44].

BMR’s sex identification in the field is generally based on genital anatomy, but in juveniles and in some cases, when only tissue or parts of dead specimens (e.g., roadkill) are available, sex identification is not possible. To address this problem, we tested *Sry* gene primers previously used for fast molecular sex identification in different mammal groups, as summarized in [45].

To address the aforementioned requirements and constraints, this study introduces a novel non-lethal karyotyping protocol based on primary fibroblast cell cultures. In addition, we propose an alternative approach for rapid monitoring—genetic methods for cryptic species identification—and present fast molecular sex determination in the blind mole rat. We further emphasize the significance of non-lethal sampling in the case study of the European lesser blind mole rat cryptic species, demonstrating that such procedures yield sufficient biological material for both, karyological and genetic analyses, without negatively impacting populations through animal sacrifice.

## 2. Materials and Methods

Total sample of 33 specimens of the five BMR cryptic species were collected during the period of 2019–2024 from 25 localities in Serbia (Figure S1 and Table S1), implementing methodology described in detail [31]. According to strictly protected species status, we developed the protocol for data collection without animal sacrificing. The Ministry of Environmental Protection of the Republic of Serbia issued annual permits that allowed the capture and sampling of the limited number of animals per location. Animals were treated in accordance with Directive 2010/63/EU of the European Parliament and of the Council of 22 September 2010 on the protection of animals used for scientific purposes. Procedures were approved by the Ethical Committee for the Use of Laboratory Animals of the Institute for Biological Research “Siniša Stanković”, University of Belgrade and the Veterinary Directorate of the Ministry of Agriculture, Forestry, and Water Economy of the Republic of Serbia (Licence No. 323-07-11307). The sampling was carried out with the assistance of a veterinarian using short-term (5–10 min) isoflurane inhalation anaesthesia. A skin biopsy was taken from a hind foot fingertip, followed by local application of 5% lidocaine topical jelly. This method of tissue sampling is particularly advantageous for experimental studies, as it allows straightforward administration of anaesthesia, rapid induction, precise regulation of anaesthetic depth, and is associated with a low incidence of complications [46]. Animals resumed normal respiration, mobility, and feeding behavior within 10 min after sampling. After a brief recovery, each animal was released back into its own underground tunnel system.

Skin tissue was used for both, the establishment of the primary fibroblasts cells cultures and for DNA extraction. Total DNA was extracted from skin biopsies and additionally from liver tissue obtained from animals that had died incidentally, either due to road accidents or predation, using the DNeasy Blood and Tissue Kit (Qiagen, Hilden, Germany).

### 2.1. Establishment of Fibroblasts Cells Cultures

Finger selected for sampling was thoroughly cleaned with 70% ethanol. After removal with sterile instruments, skin biopsy was first washed briefly in 70% ethanol for 2 min, then rinsed four times in sterile saline solutions containing antibiotics (penicillin 100,000 U/L and streptomycin 100 mg/L) and an antimycotic (amphotericin B 25 μg/mL) 2 h in total. After incubation in 0.5% trypsin with 0.2% EDTA at 37 °C for 30 min, the skin sample was transferred to a tube containing complete medium (RPMI growth medium with L- Glutamine, 10% of fetal bovine serum (FBS) and 1% antibiotics/antimycotic) to block the effect of trypsin. Subsequently, it was transferred to Petri dish for mechanical shredding using sterile scissors and a scalpel. The crushed pieces are transferred and resuspended in 1–2 mL of collagenase solution (1 mg/mL) dissolved in complete medium and tissue was incubated at 37 °C in thermoshaker at 250 rpm overnight.

Additionally, the effectiveness of three different types of collagenase in establishing BMR’s fibroblast cell culture was tested: Collagenase type I (Gibco™, Waltham, MA, USA), Collagenase Type II and type IV (Merck, Darmstadt Germany). For this purpose additional fibroblast cell culture was set from skin biopsy of laboratory Wistar rat for comparison with BMRs. The tissue sample is then centrifuged for 7 min and the supernatant is removed. The sample is resuspended in 2 mL complete medium and passed through a cell strainer (mesh size 70 μm). This final suspension is placed in a T25 flask and complete medium is added to a final volume of 3 mL.

Primary fibroblasts cells were cultivated in CO_2_ controlled incubator (5% CO_2_) at a temperature of 37 °C, in complete RPMI growth medium with L- Glutamine contained 10% of fetal bovine serum (FBS).

Cell growth was monitored in different phases of proliferation and photographed using ZOE Fluorescent Cell Imager (Bio-Rad, Hercules, CA, USA). The medium was changed every four days and additional FBS was periodically added when cells exhibited slow proliferation. A cell passage, using a solution of 0.05% trypsin and 0.05% EDTA in a 1:1 ratio, was performed each time the cells completely covered the surface of the flasks (confluence > 80%). After a few passages, the quantity of cells was sufficient for chromosome preparation.

### 2.2. Chromosome Preparation and Karyotyping

At least two completely covered T25 flasks were used for chromosome preparations. Colchicine was added at a final concentration of 0.016 μg/mL, after which they were incubated for a further 24 h, then ethidium bromide was added at a final concentration of 2 μg/mL. After three hours, cells were removed by standard trypsinisation, transferred to a 12 mL test tube, and treated with hypotonic solution in a water bath at 37 °C. Optimization of hypotonic treatment for BMR chromosome preparations included different solutions and treatment times (30 to 60 min): 7.75 mM potassium chloride; 0.9% sodium citrate; a combination of 33.5 mM potassium chloride with 7.75 mM sodium citrate (1:1); a combination of 41 mL of 0.56% potassium chloride solution, 8 mL of distilled water and 1 mL of fetal bovine serum (FBS); and a mixture of distilled water and complete medium in a ratio of 3:1.

Cells were prefixed with fresh cold fixative (methanol and glacial acetic acid in ratio 3:1), centrifuged 5 min at 1700 rpm, and incubated 12 min at 4 °C. Supernatant was discharged and 3 mL of ice-cold fixative was carefully added to the pellet without mixing and incubated 30 min at −20 °C. After the second round of fixation, a few drops of the chromosome suspension were dropped from a height of approximately 20–30 cm onto a clean wet microscope slide and left to dry in an upright position for a few hours. At least 20 conventionally Giemsa stained metaphases per individual were analysed. Ideograms were obtained when needed as an additional tool for defining chromosomal morphology and calculating NF, using the software KaryoMeasure ver. 1.7.5 [47]. Cryptic species were identified from obtained karyotypes and idiograms following [36].

For fibroblast cell culture-based karyotyping in this study, we used a subsample of eight specimens, previously identified by DNA barcoding, which belong to different cryptic-species (Table S1).

### 2.3. Cryptic Species Identification

Twelve ISSR primers used previously for identification of mice species [42,48] were optimised for identification of five BMR cryptic species, and primers that produced DNA profiles of moderate complexity with a clear band pattern and the presence of species-specific fragments were chosen. Amplifications were carried out in a final volume of 20 μL following the procedures described in [48]. Reactions were performed under the following thermal conditions: initial denaturation 5 min at 94 °C, followed by 45 cycles (30 s at 94 °C; annealing temperature (Ta) 30 s at 48 °C to 56 °C for different primers; 90 s at 72 °C) and a final extension 8 min at 72 °C in a Thermal Cycler 2720 (Applied Biosystems, Foster City, CA, USA). PCR products were separated by horizontal gel electrophoresis (1.25% agarose; 20 μL of each PCR product) for 100 min at 120 V per electrophoretic run. Standard molecular weight marker (GeneRuler 100 bp DNA Ladder Plus, Fermentas, Vilnius, Lithuania) was used. The bands were visualised under UV light using the Bio-Rad Gel Doc XR+ System, and the results were examined using GelAnalyzer 19.1 [49].

To confirm ISSR-PCR results, i.e., the affiliation to a particular cryptic-species, ISSR profiles of a total of 31 BMR individuals were compared with cyt *b* sequences.

### 2.4. Sry Sex Determination

For sex identification of European BMRs, we tested three different primer pairs (SRY-A, SRY-B, and SRY-HMG box) targeting different regions of the *Sry* gene, which have previously been tested in different mammal species [50,51,52,53]. In all cases, a *Zfy-Zfx* region was co-amplified as a positive control for a successful PCR reaction [53]. All PCR reactions for the primers listed in Table 1 were carried out in a final volume of 25 μL, following the procedures described in the respective studies.

## 3. Results

### 3.1. Establishment of Fibroblast Cells Cultures

**Figure 1:**
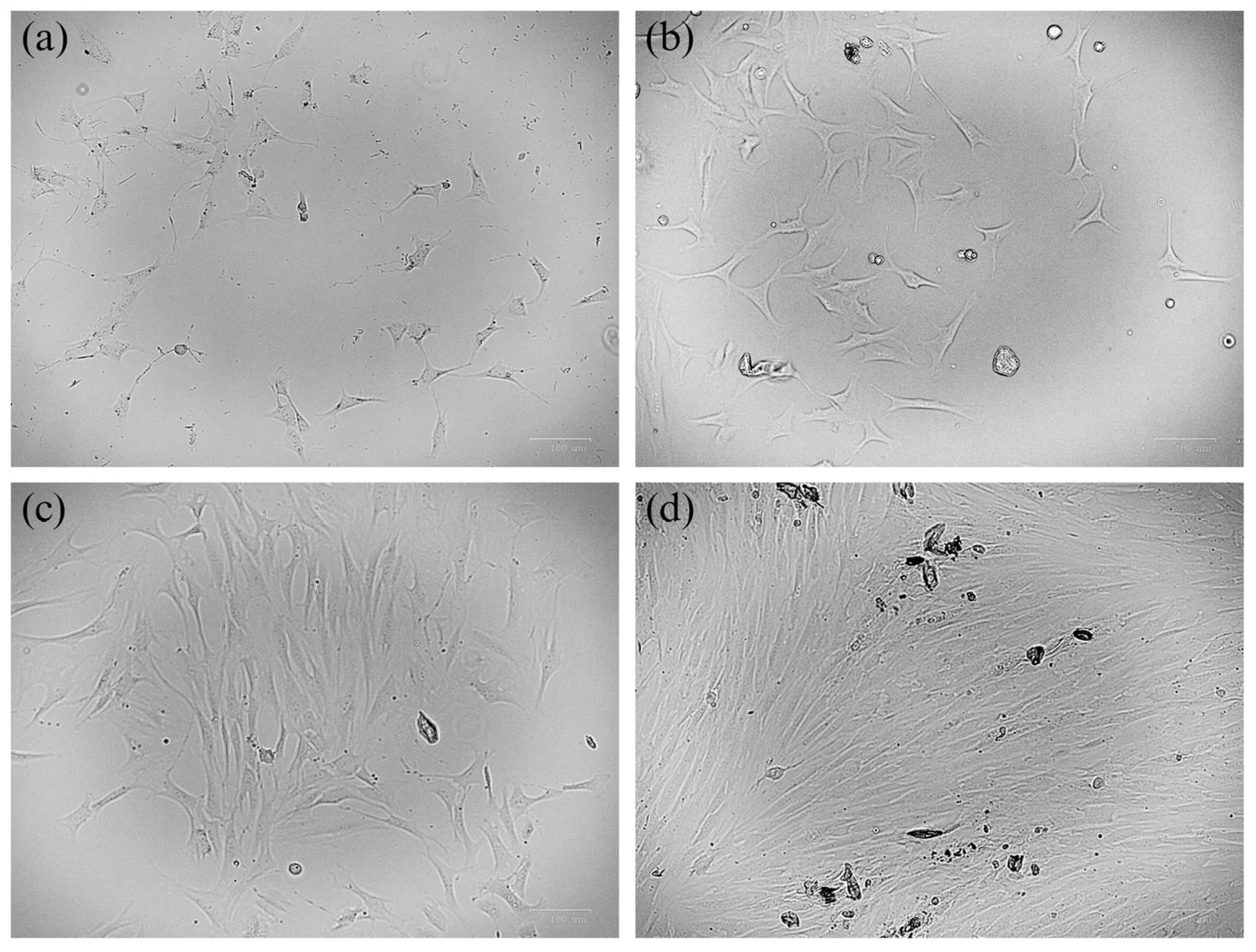
A section of BMRs fibroblasts in different phases of proliferation photographed with the ZOE Fluorescent Cell Imager at 10× magnification. (**a**) Early stage of primary fibroblast culture showing individual fibroblast cells diffusely distributed across the culture surface; (**b**) after one week of cultivation, fibroblasts have proliferated and established extensive cell-to-cell contacts, forming an interconnected cellular network; (**c**) fibroblast cells in the exponential growth phase; (**d**) near-confluent fibroblast monolayer suitable for chromosome preparation.

For in vitro cultivation and expansion of BMR fibroblast cell cultures, one of the critical initial steps was correct tissue digestion, i.e., application of the appropriate type of collagenase. While rat control cell growth was initially reduced by half with collagenase type IV and after 2–3 weeks eventually reached exponential growth, BMR’s cell growth was extremely dependant on collagenase type. Namely, stable cultures were established only when type I collagenase was used. Following collagenase II and IV digestion, BMR cultures yielded scant fibroblast cells that exhibited no further proliferation and underwent cell death. Primary cultures supplemented with additional FBS exhibited significantly faster and more successful cell growth. The cultured fibroblast cells achieved cellular confluence and amount necessary for karyotypisation within a month (Figure 1).

### 3.2. Chromosome Preparation and Karyotyping

Out of all combinations of hypotonic solutions, only a mixture of distilled water and complete medium in a 3:1 ratio at 37 °C for 1 h was successful for karyotyping. To avoid chromosomal aberrations caused by cryopreservation and repeated passages, we used fibroblasts from no more than four passages. The karyotypes and idiograms, acquired from at least 20 examined metaphases per specimen, were clearly differentiated. Chromosomes were classified according to their morphology into four groups: metacentrics, submetacentrics, acrocentrics, and subacrocentrics.

**Figure 2:**
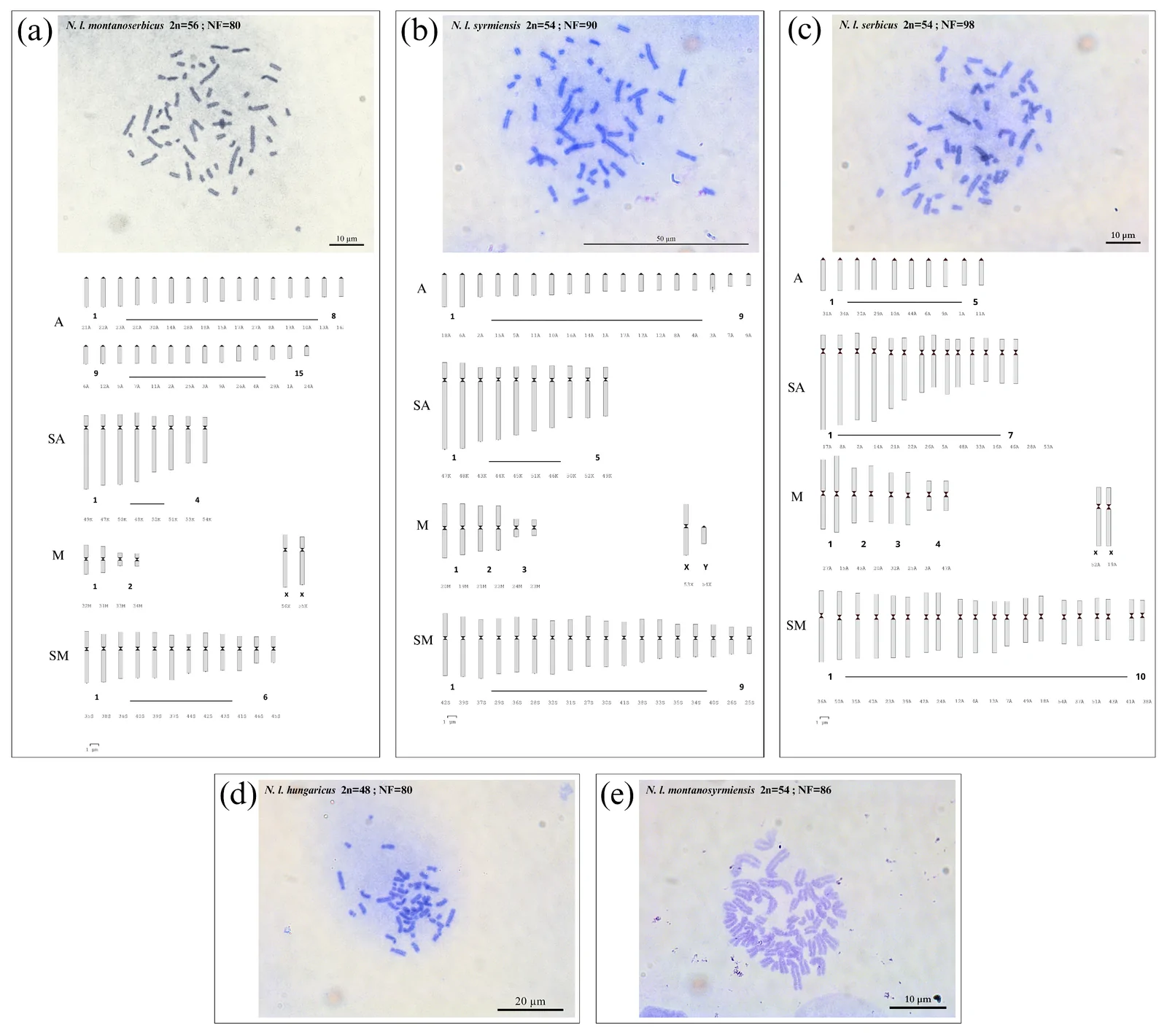
Giemsa-stained karyotypes (**top**) and idiograms (**bottom**) presenting different 2n and chromosome morphology. A—acrocentrics; SA—subacrocentrics; M—metacentrics; SM—submetacentrics; (**a**) a female *N. l. montanoserbicus* (2n = 56; NF = 80); (**b**) a male *N. l. syrmiensis* (2n = 54; NF = 90), (**c**) a female *N. l. serbicus* (2n = 54; NF = 98); (**d**) a male *N. l. hungaricus* (2n = 48; NF = 80); (**e**) a female *N. l. montanosyrmiensis* (2n = 54; NF = 86).

Metaphase chromosome spreads of each cryptic species (2n from 48 to 56) and idiograms of the two evolutionary closest *N. l. syrmiesnis* and *N. l. serbicus*, together with the species with the highest 2n (*N. l. montanoserbicus*) are presented in Figure 2.

*N. l. montanoserbicus* exhibited the highest 2n = 56, with two pairs of metacentric autosomes (the first medium-sized and the second among the smallest chromosomes in the karyotype), six pairs of submetacentric autosomes, four pairs of subacrocentrics, and 15 pairs of acrocentics (Figure 2a)*. N. l. syrmiensis* karyotype comprises three pairs of metacentrics, nine pairs of submetacentrics, five pairs of subacrocentrics (the longest chromosomes in the karyotype), and nine pairs of small acrocentric autosomes (Figure 2b). Unlike to *N. l. syrmiesis*, *N. l. serbicus* exhibited lower number of acrocentrics (only five pairs) (Figure 2c), four pairs of metacentrics, 10 pairs of submetacentrics, and seven pairs of subacrocentric chromosomes.

Individuals with a diploid chromosome number of 2n = 48 (Figure 2d) were identified as *N. l. hungaricus* with four pairs of large metacentrics, eight pairs of submetacentrics, five pairs of long subacrocentrics (mostly the largest chromosomes in the karyotype) and six pairs of acrocentrics. Finally, *N. l. montanosyrmiensis* 2n = 54 (Figure 2e) has two pairs of medium-sized metacentrics, eight pairs of submetacentrics, five pairs of long subacrocentrics, and 11 pairs of small acrocentric chromosomes.

### 3.3. Cryptic Species Identification Using ISSR-PCR

Of the 12 primers optimised here for BMR cryptic species identification, the three most informative were selected for analysis of 33 samples in total. Primers P5, P3, and P10 produced moderately complex ISSR profiles with species-specific diagnostic bands. The primer sequences and specific Ta are presented in Table 1. Repeatability and reproducibility were confirmed by comparing ISSR profiles among three replicates. Only clear, unambiguous, and reproducible bands, consistently detected across samples and independent PCR reactions, were scored as informative ISSR markers.

**Figure 3:**
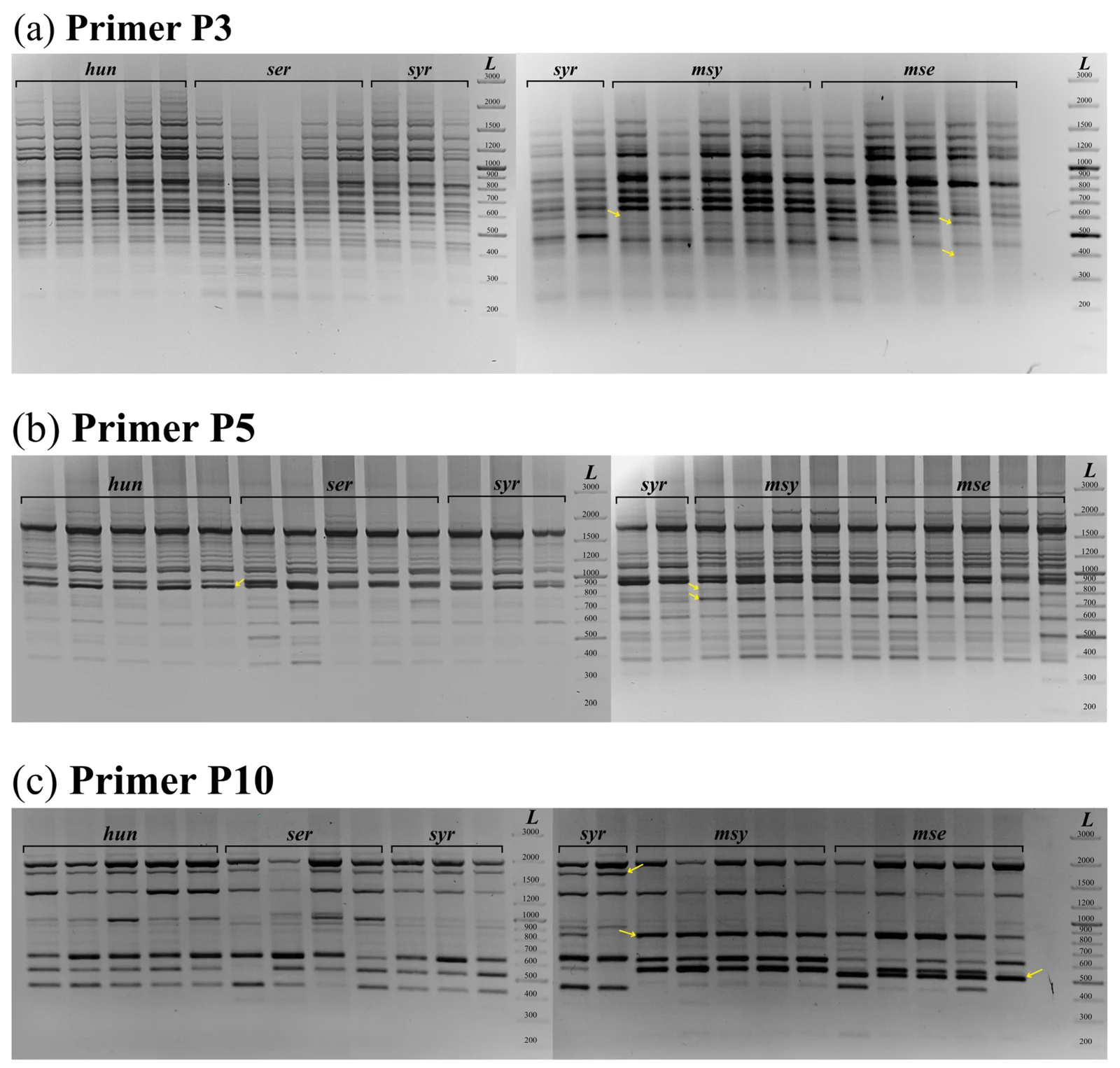
Agarose gel electropherograms of ISSR-PCR profiles generated using primers P3 (**a**), P5 (**b**), and P10 (**c**) with species-specific band patterns. *N. l. hungaricus* (hun); *N. l. serbicus* (ser); *N. l. syrmiensis* (syr); *N. l. montanosyrmiensis* (msy); and *N. l. montanoserbicus* (mse). L—GeneRuler 100 bp Plus DNA Ladder (Fermentas, Vilnius, Lithuania) (bp). Yellow arrows indicate distinctive species-specific ISSR bands.

ISSR profiles produced by P3 primer were the most complex, with more than 30 bands (Figure 3a). This primer was applicable for differentiation between phylogenetically early diverged “montano” clade from the group of more recently diverged clade *N. l. hungaricus*, *N. l. serbicus*, and *N. l. syrmiensis*, as well as to distinguish “montano” species from each other. The *N. l. montanosyrmiensis* ISSR fingerprint was recognisable by the absence of 587 bp and 431 bp bands, detected in the ISSR profiles of other taxa. In contrast, the *N. l. montanoserbicus* ISSR fingerprint was characterised by missing 418 bp band, observed in all four cryptic species, including *N. l. montanosyrmiensis*. The evolutionarily younger *N. l. hungaricus*, *N. l. serbicus*, and *N. l. syrmiensis* ISSR profiles shared common DNA bands, without a species-specific band pattern.

Primer P5 produced a 766 bp band diagnostic for both “montano” cryptic species and an additional 848 bp species-specific band for *N. l. montanosyrmiensis*. This marker was the most informative, as it allowed differentiation of one species from three evolutionary younger species, with an 812 bp band species-specific for *N. l. hungaricus* (Figure 3b).

The P10 primer generated an ISSR profile of moderate complexity, enabling easy identification of *N. l. montanoserbicus* and *N. l. montanosyrmiensis* from other cryptic species (Figure 3c). The ISSR-profiles of both cryptic species exhibited a distinguished band pattern, characterised by the absence of the 1750 bp band and the presence of highly intense 825 bp and 540 bp bands. *N. l. montanoserbicus* had a species-specific 520 bp band, which was not observed in the ISSR profile of all other cryptic species. *N. l. syrmiensis*, *N. l. hungaricus*, and *N. l. serbicus* shared common ISSR bands, although with different individual quantity. Two *N. l. serbicus* samples from the Niš population exhibited a population-specific 1003 bp band.

Confirmation of ISSR-PCR results: a total of 31 specimens analysed here were additionally identified by cyt *b* barcoding: 18 samples were taken from our previous publications and 13 were newly sequenced in this study (GenBank accession numbers are listed in Table S1). They included: nine specimens of *N. l. syrmiensis*; six of, *N. l. serbicus*, and *N. l. hungaricus*, and five of *N. l. montanoserbicus* and *N. l. montanosyrmiensis.* Finally, ISSR-PCR method allowed discrimination of four out of five cryptic species.

### 3.4. Sry Sex Determination

**Figure 4:**
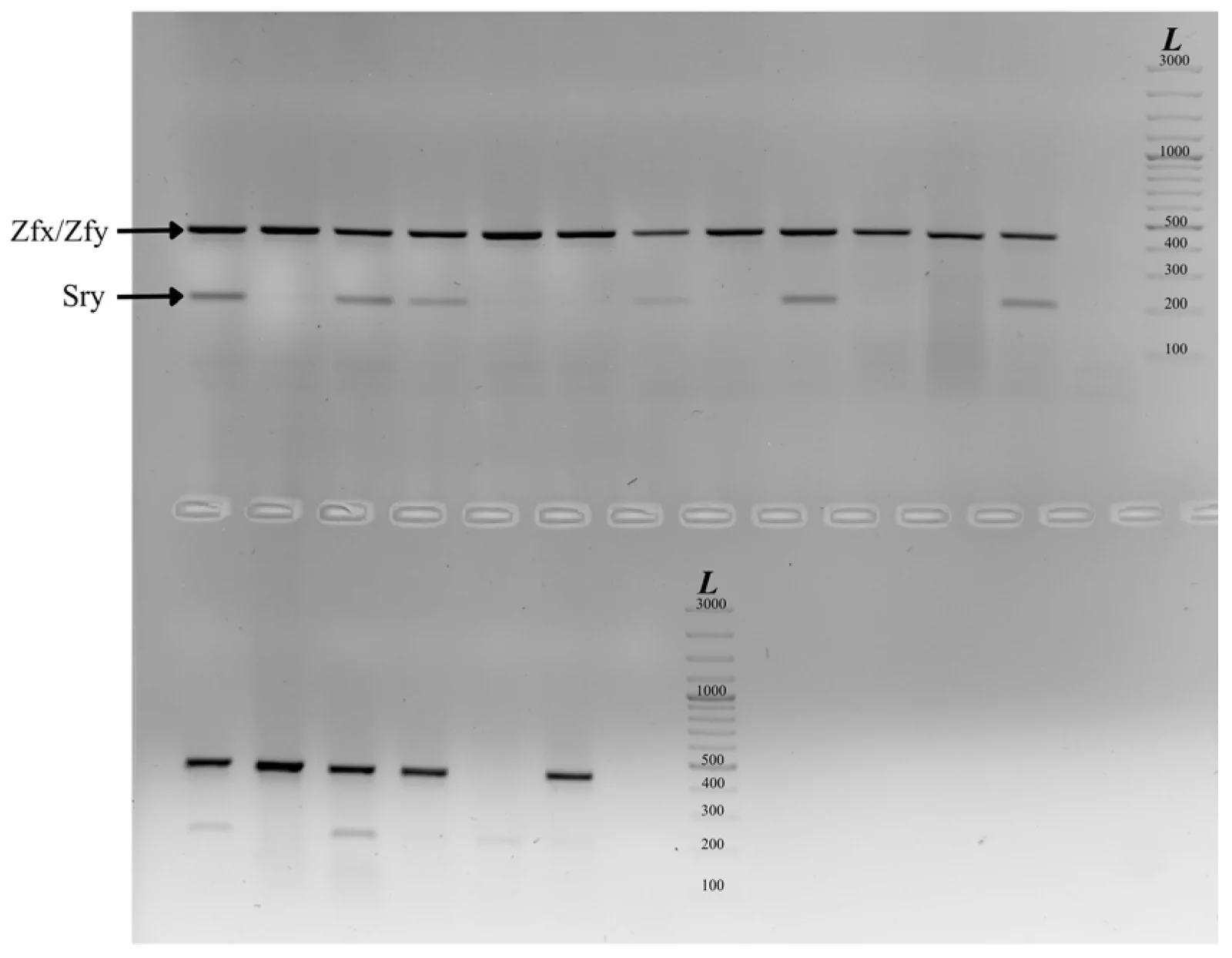
PCR products amplified using *Sry-HMG* box primers, visualized on 1.2% agarose gel Black arrows indicate: 445 bp Zfx/Zfy internal control and 202 bp *Sry*-specific fragment. L—GeneRuler 100 bp Plus DNA Ladder (Thermo Scientific™).

Out of three tested *Sry* primer pairs, *Sry-HMG* box primers proved to be accurate for sex identification of European BMR individuals. PCR mix contained 0.3 µL of each of 20 µM Zfy-Zfx control primers and 0.7 µL of 20 µM of each of SRY primers, as well as 5 µL of DNA template (50 ng/µL) in 25 µL of the final volume. Temperature profile: initial denaturation 94 °C for 3 min; 30 cycles (94 °C for 1 min, 50 °C for 1 min, 72 °C for 1 min) and a final elongation 72 °C for 7 min. Samples exhibiting both the 445 bp *Zfy-Zfx* control band and the 202 bp *Sry* band were scored as males, while those showing only the 445 bp control band were scored as females (Figure 4).

## 4. Discussion

Accurate biodiversity assessment and effective conservation planning require species identification and delimitation, especially for endemic and threatened species [54]. Reliable species identification is therefore a critical first step in conservation. Misidentification can compromise conservation efforts, as protection may be directed towards the wrong populations, while genuinely threatened taxa remain overlooked.

While traditional morphological taxonomy remains indispensable for museum curation, field identification, and nomenclatural stability, morphology-only approaches often fail when confronted with cryptic species complexes, in which distinct evolutionary lineages exhibit little or no diagnostic external differences. In such cases, reliance on morphology alone leads to persistent underestimation of diversity and misinformed conservation priorities [5]. Thus, an integrative taxonomic framework that combines chromosomal, molecular, morphological, and geographic evidence (as exemplified within the Anatolian *Nannospalax* complex proposed by [35] provides the most robust and reliable basis for species delimitation.

For genetic monitoring workflow—the identification and delimitation of morphologically indistinguishable cryptic species, we developed non-lethal molecular genetic and cytogenetic tools, by drawing on our long-term experience with protected and threatened European lesser BMR. Our study demonstrates that a non-lethal, comprehensive approach, that combines karyotype analysis, ISSR-PCR species detection and molecular *Sry* sex determination, provides a pathway for species identification and delimitation and represents a reliable first step in the conservation of the *Nannospalax leucodon* species complex. ISSR-PCR represents a rapid and cost-effective method particularly well suited for large-scale monitoring, as it enables the analysis of numerous individuals and populations across extensive geographic regions. At the same time, non-lethal karyotype analysis remains indispensable for chromosomal species—cryptic taxa characterized by pronounced chromosomal plasticity—where molecular genetic identification must be complemented by detailed cytogenetic examination [35]. In cases where morphological sex identification is difficult or impossible, molecular *Sry* gene determination provides a solution.

Karyotypes, described by chromosome number and morphology, are typical phenotypic traits that can distinguish reproductively isolated units [3]. In addition, karyotype analyses can reveal chromosomal evidence of hybridisation events between distinct taxa, information that is often undetectable using the mitochondrial DNA barcoding approaches traditionally employed for species identification. In European BMRs, chromosomal forms have initially been described according to karyotypes [55,56] and used as the primary basis for cryptic species recognition [28,35]. Therefore, karyotype analysis was mandatory in this study, in order to link molecular genetic identification with the originally described chromosomal forms.

Our main focus was the development of a karyotyping method that is safe for animals. All previous karyotyping methods for the BMR were based on chromosome preparations from bone marrow, which necessitated sacrificing the animals. Moreover, reports on the establishment of blind mole rat cell cultures have been contradictory: earlier reports suggested that fibroblast cultures of BMRs could not persist for extended periods in cell culture due to exceptional concerted cell death, i.e., their unique cellular mechanisms linked to programmed cell death [37].

Although fibroblast cell cultures have previously been established in *Spalax ehrenbergi* for investigations of anti-cancer mechanisms [57], their use for karyotyping, particularly in European BMRs, has not been reported. To the best of our knowledge, our study provides the first demonstration of fibroblast cell culture-based karyotyping in BMRs without affecting animals survival. Here we demonstrate that cell cultures can be successfully established and cultivated for a long time by following certain recommendations, primarily due to their high sensitivity to collagenase type. Stable growth was achieved exclusively with collagenase type I tissue digestion, while other types reduced the cells’ adhesive capacity. Furthermore, BMR fibroblasts proliferated approximately four times more slowly than rat controls and frequently required additional FBS supplementation. The comparison of the obtained karyotypes and their concordance with those described earlier provides strong support for the reliability of karyotype identification of cryptic species based on a non-lethal approach. Namely, the karyotype of each specimen was completely concordant with the karyotype of the appropriate cryptic species previously published [27,36,58].

By successfully establishing fibroblast cell cultures from non-lethal sampling, we overcame the ethical and methodological limitations of bone marrow-based karyotyping. We therefore propose this method as a practical monitoring tool, especially for taxa with high chromosomal diversity. This protocol enables reliable identification and delimitation of cryptic species and chromosomal forms, both of which are potentially important conservation units. Moreover, this method has the potential to be applied to other cryptic and strictly protected taxa as part of an integrative framework for species recognition. In contrast, not considering karyotypes and using a single methodological approach in recognizing chromosomally diverse taxa [59] may result in incomplete species delimitation and the erroneous lumping of multiple cryptic species into a single taxonomic entity, which can ultimately compromise their conservation and long-term survival.

Traditionally, DNA barcoding—comparison of mitochondrial DNA gene sequences such as cytochrome *b*, *16S rRNA*, cytochrome *c* oxidase subunit I (*COI*), and NADH dehydrogenase subunit 1—is widely used for species identification [60,61,62,63]. However, standard DNA barcoding can be insufficient to distinguish recently diverged sister species due to low differentiation or high polymorphism among these markers [64]. Moreover, sequencing generally requires several days and additionally, costs can still be a limiting factor, particularly in large-scale monitoring with large sample sizes or in less developed regions.

Because of their exceptionally high mutation rates, microsatellites, or simple sequence repeats (SSR), can in some cases be more informative than other types of DNA markers [65,66,67], and an alternative approach, ISSR-PCR may serve as a valuable complementary tool, particularly when standard DNA barcoding fails to distinguish closely related or sister species, since specific ISSR markers can occasionally reveal diagnostic differences. The assay enables species identification within a few hours using a single PCR reaction, making it particularly suitable for large-scale monitoring that requires rapid screening of vast numbers of animals. ISSR-PCR generates reproducible dominant markers without prior knowledge of the DNA template [40,43,44]. ISSR primers are broadly applicable across a wide range of taxa [68,69], generating a large number of loci across the whole genome. ISSR markers have been successfully used in the identification of plant [70], fish [41], rodent [42], and bat species [43], and are now successfully applied for the identification of BMR cryptic species. We show that three ISSR-PCR primers delineate four out of five cryptic BMR species distributed in Serbia. Comparison of ISSR profiles with karyotypes and cyt *b* sequences confirmed that the ISSR-PCR method correctly assigned each individual to appropriate cryptic species. ISSR patterns were identical for all individuals of the same cryptic species from different populations. Using the appropriate primer, we were able to distinguish early-diverged “montano” groups (*N. l. montanoserbicus* and *N. l. montanosyrmiensis*) from more recently diverged cryptic BMR species (*N. l. hungaricus*, *N. l. serbicus*, and *N. l. syrmiensis*) [30,31,71]. The resolution of ISSR-PCR–based species identification was lower for evolutionarily younger and recently diverged taxa, particularly for the two most closely related lineages, *N. l. serbicus* and *N. l. syrmiensis* [31,71], between which no diagnostic ISSR-PCR differences were detected. Further testing of the newly designed ISSR primers will be necessary to develop an assay that produces clear band patterns, enabling delineation among *N. l. serbicus* and *N. l. syrmiensis.* Despite the lack of a diagnostic ISSR primer, these two cryptic species could not be misidentified in the field, as their distribution areas are naturally separated by the Great Morava River [55]. Although ISSR markers did not fully resolve all taxa, their concordance with sequencing and karyotyping results highlights their utility as part of an integrated diagnostic framework. Practical application of the ISSR-PCR method focuses on the rapid delineation of cryptic species that may be sympatrically distributed (*N. l. montanosyrmiensis* and *N. l. hungaricus* in the Vojvodina region; *N. l. montanoserbicus* and *N. l. sebicus* in southern Serbia), allowing identification within a few hours, using a single PCR. Thus, the ISSR-PCR method does not replace other established methods such as DNA barcoding for cryptic species identification and delimitation, but it can offer clear advantages in speed, cost, minimal equipment requirements, and practicality for processing large sample sets.

Additionally, *Sry-HMG* box primers previously successfully applied for sex determination in other rodent species [45] were also effective in BMRs. This further supports the value of non-lethal sampling approaches, particularly in cases where morphological sex determination is not possible. This method proved especially valuable for further planned BMR morphometric analysis, as it was shown recently that morphological delineation based on cranial size and overall shape data between these cryptic species must be carried out separately for each sex because of pronounced sexual dimorphism [32].

The European lesser BMR, *N. leucodon* superspecies, represents a complex of species and subspecies [72] and is currently listed in the IUCN Red List as Least Concern (LC) at both global and European level and as Data Deficient (DD) at the Mediterranean level, with a notion that taxonomy is unresolved [73]. Historically, the taxon was classified as Vulnerable until 1996, and subsequently as DD due to persistent difficulties in distinguishing morphologically similar cryptic species. Of 25 reported chromosomal forms, seven have been demonstrated to be reproductively isolated [28] and clear molecular genetic evidence further supports their recognition as distinct species [29,30,31,71]. In this context, the incomplete species designation combined with the assignment of a “Least Concern” status may represent a setback for effective conservation of European BMRs. Although the entire superspecies is strictly protected in Serbia, two cryptic species are currently assessed as Critically Endangered under criteria B1ab(iii,v) and B2ab(iii,v) [31,71].

It is also important to note that cryptic species of the lesser BMR in Serbia should not be regarded as subspecies; rather, they represent distinct species that currently lack morphological diagnostic criteria and therefore formal recognition under ICZN rules. However, the use of binomial classification, such as *Nannospalax syrmiensis*, is not supported by the rules of zoological taxonomy, and such names would be considered *nomina nuda*; therefore, we currently use trinomial classification (e.g., *Nannospalax leucodon syrmiensis*). What remains to be satisfied are the principles of classical taxonomy, specifically the identification of morphological differences between species. The initial and essential step that addressed the question of sexual dimorphism [32] allowed morphometric analysis, which combined with karyotype characterization and molecular genetic approaches, enable an integrative framework for the identification, delimitation, and formal description of species, while also providing a more realistic insight into their evolutionary relationships. Thus, the non-lethal framework presented here could be a valuable component of this integrative approach, providing cytogenetic and molecular data without the need to sacrifice individuals. Such strategy could facilitate more accurate species delimitation, improve the recognition of conservation units, and ultimately support more effective conservation and management actions.

## 5. Conclusions

This study introduces the first integrated non-lethal framework for cytogenetic and molecular monitoring of the *Nannospalax leucodon* species complex. By combining fibroblast culture-based karyotyping, applied here for the first time to blind mole rats, with ISSR-PCR species identification, and *Sry*-based sex determination, we established a reliable approach for identifying and monitoring cryptic species without sacrificing individuals. It is also important to emphasize that, for phylogenetically closely related or cryptic species, testing a broader set of anchor ISSR primers is necessary to generate species-specific discrepancies. We will further apply this framework in our ongoing research to obtain a comprehensive overview of chromosomal diversity across different populations. Beyond its application to European BMRs, this framework provides a practical tool for conservation monitoring of protected species, particularly where traditional sampling is ethically or legally constrained. Species hypotheses corroborated by multiple lines of evidence are less likely to reflect artifacts of sampling or marker choice, thereby providing a more reliable foundation for red-listing, legal protection, and management decisions. Within this context, the proposed framework serves as a valuable instrument for conservation programmes and advances an integrative taxonomy approach essential for the study and protection of cryptic taxa.

## Figures and Tables

**Table 1:** The primer sequences and specific annealing temperature (Ta) of all optimized primers; fw—forward primer; rev—reverse primer.

Primer Name	Primer Sequence	Ta (°C)	Reference
P3	5′-(AG)_8_-3′	56	[48]
P5	5′-(CAA)_5_GC-3	48	[48]
P10	5′-(GA)_8_AC-3′	56	[48]
SRY-HMG fw *	5′-GTCAAGCGCCCCATGAATGCAT-3′	50	[52]
SRY-HMG rev *	5′-AGTTTGGGTATTTCTCTCTGTG-3′		[52]
SRYA-5	5′-TGAACGCATTCATGGTGTGGT-3′	54	[50]
SRYA-3	5′-AATCTCTGTGCCTCCTGGAA-3′		[50]
SRYB-5	5′-TGAACGCTTTCATTGTGTGGT C-3′	55	[50]
SRYB-3	5′-GCCAGTAGTCTTGTGCCTCCT-3′		[50]
Zfy-Zfx fw *	5′-ATAATCACATGGAGAGCCACAAGCT-3′	50	[53]
Zfy-Zfx rev *	5′-GCACTTCTTTGGTATCTGAGAAAGT-3	50	[53]

* Abbreviated names of the forward and reverse primers.

## Data Availability

DNA nucleotide sequences were deposited in the NCBI GenBank, and can be assessed at https://www.ncbi.nlm.nih.gov/genbank, accessed on 26 February 2024.

## Supplementary Materials

The following supporting information can be downloaded at: https://www.mdpi.com/article/10.3390/life16091408/s1, Figure S1: Map showing the localities of specimens included in this study; Table S1: List of blind mole rat samples analysed in this study, with corresponding cytochrome *b* (cyt *b*) GenBank accession numbers (ID); RS—Republic of Serbia.
