# Supplementary material for: Methodological Perspectives on Cryptic Species Monitoring: Insights from Strictly Protected Lesser Blind Mole Rat
Source: Life (Basel). 2026 Aug 25;16(9):1408. doi: 10.3390/life16091408 (PMC13608712; doi:10.3390/life16091408)
Supplement: Supplementary file 1 [file life-16-01408-s001.zip › Table S1.pdf]

Table S1. List of blind mole rat samples analysed in this study, with corresponding cytochrome *b* (cyt *b*) GenBank accession numbers (ID); RS – Republic of Serbia.

No	Sample No	Cryptic species/ sex	Sampling locality	Cyt *b* ID	Reference	Method
1.	1RS	*hungaricus*/male	Šumarak, RS	OM714859	Bugarski-Stanojević et al., 2020	ISSR, *Sry*
2.	4RS	*hungaricus*/ female	Pančevo, RS	OM714862	Bugarski-Stanojević et al., 2020	ISSR, *Sry*
3.	5RS	*hungaricus*/ male	Kajtasovo, RS	OM714863	Bugarski-Stanojević et al., 2020	ISSR, *Sry*
4.	22RS	*serbicus*/ female	Klinovac, RS	OM714871	Bugarski-Stanojević et al., 2020	ISSR, *Sry*
5.	40RS	*syrmiensis*/ female	Beograd, RS	OM714883	Bugarski-Stanojević et al. 2022	ISSR, *Sry*
6.	43RS	*hungaricus*/ male	Vršac, RS	OM714886	Bugarski-Stanojević et al. 2022	ISSR, *Sry*
7.	62RS	*montanosyrmiensis*/ female	Sremski Karlovci, RS	OM714893	Bugarski-Stanojević et al. 2022	ISSR, *Sry*
8.	64RS	*montanoserbicus*/ male	Vlasina, RS	OM714895	Bugarski-Stanojević et al. 2022	ISSR, *Sry*
9.	76RS	*montanosyrmiensis*/ male	Krušedol, RS	PZ797853	This study	ISSR, *Sry*
10.	78RS	*montanosyrmiensis*/ male	Stražilovo, RS	PZ797854	This study	ISSR, *Sry*
11.	79RS	*montanosyrmiensis*/ male	Stražilovo, RS	PZ797855	This study	ISSR, *Sry*
12.	87RS	*syrmiensis*/ male	Košutnjak, RS	PP378191	Bugarski-Stanojević et al. 2024	ISSR, *Sry*
13.	89RS	*serbicus*/ female	Niš, RS	PP378181	Bugarski-Stanojević et al. 2024	ISSR, *Sry*
14.	90RS	*serbicus*/ male	Niš, RS	PP378182	Bugarski-Stanojević et al. 2024	ISSR, *Sry*
15.	91RS	*syrmiensis*/ male	Vinča, RS	PP378192	Bugarski-Stanojević et al. 2024	Karyotyping, ISSR
16.	92RS	*syrmiensis*/ male	Vinča, RS	PP378193	Bugarski-Stanojević et al. 2024	ISSR, *Sry*
17.	93RS	*syrmiensis*/ unknown	Udovice	PP378194	Bugarski-Stanojević et al. 2024	Karyotyping, ISSR
18.	94RS	*syrmiensis*/ male	Lešće, RS	PP378195	Bugarski-Stanojević et al. 2024	Karyotyping, ISSR
19.	97RS	*serbicus*/ female	Ristovac, RS	PP378183	Bugarski-Stanojević et al. 2024	ISSR, *Sry*
20.	103RS	*hungaricus*/ male	Deliblato, RS	PZ797856	This study	Karyotyping, ISSR
21.	107RS	*montanosyrmiensis*/ female	Krušedol, RS	PZ797857	This study	ISSR, *Sry*
22.	108RS	*montanoserbicus*/ female	Ševarice, RS	PP378186	Bugarski-Stanojević et al. 2024	ISSR, *Sry*
23.	111RS	*montanoserbicus*/ female	Ševarice, RS	/	This study	Karyotyping
24.	112RS	*montanoserbicus*/ male	Ševarice, RS	PP378187	Bugarski-Stanojević et al. 2024	ISSR, *Sry*
25.	114RS	*syrmiensis*/ male	Leštane, RS	PZ797858	This study	Karyotyping, ISSR, *Sry*
26.	117RS	*syrmiensis*/ female	Ritopek, RS	PZ797865	This study	ISSR
27.	118RS	*syrmiensis*/ female	Ritopek, RS	PZ797859	This study	ISSR
28.	120RS	*serbicus*/ female	Kostolac, RS	PZ797860	This study	Karyotyping, ISSR, *Sry*
29.	125RS	*serbicus*/ male	Rogljevo, RS	PZ797861	This study	ISSR, *Sry*
30.	127RS	*hungaricus*/ female	Banatska Palanka, RS	PZ797862	This study	ISSR, *Sry*
31.	139RS	*montanoserbicus*/ female	Tara, RS	PZ797863	This study	ISSR, *Sry*,
32.	142RS	*montanoserbicus*/ female	Sjenica, RS	PZ797864	This study	ISSR, *Sry*
33.	162RS	*montanosyrmiensis*/ female	Stražilovo	/	This study	Karyotyping, *Sry*
